# Multidisciplinary mHealth Rehabilitation for Patients With Abdominal Cancer Who Are Receiving Chemoradiotherapy: Randomized Phase II Trial

**DOI:** 10.2196/98330

**Published:** 2026-09-08

**Authors:** Lei Yang, Wenbo Wang, Jing Su, Yudi Xiong, Han Wu, You Wang, Jing Dai, Dedong Cao, Jin Peng, Ling Xia, Huangang Jiang, Hui Xu, Qingyun Wang, Fengxia Chen, Qing Shu, Chizi Hao, Ke Lv, Weihu Shang, Honghong Xu, Ying Zhang, Weidong Chen, Ximing Xu, Hanping Shi, Fuxiang Zhou

**Affiliations:** 1 Department of Radiation and Medical Oncology, Hubei Key Laboratory of Tumor Biological Behaviors, and Hubei Cancer Clinical Study Center, Zhongnan Hospital of Wuhan University, Donghu Road 169, Wuhan, 430071, China, 86 02767812592; 2 Department of Radiation Oncology, Hubei Cancer Hospital, Wuhan, Hubei, China; 3 Cancer Center, Renmin Hospital of Wuhan University, Wuhan, Hubei, China; 4 Department of Rehabilitation Medicine, Zhongnan Hospital of Wuhan University, Wuhan, Hubei, China; 5 Wuhan Sports University, Wuhan, Hubei, China; 6 Beijing Ainst Medical Technology Co., Ltd, Beijing, China; 7 Health Science Center, Peking University, Beijing, Beijing, China; 8 Department of Clinical Nutrition, Beijing Shijitan Hospital, Capital Medical University, Beijing, China; State Market Regulation, Key Laboratory of Cancer FSMP for State Market Regulation, Beijing, China

**Keywords:** multimodal rehabilitation, mobile health, multidisciplinary team, abdominal cancer, concurrent chemoradiotherapy, handgrip strength

## Abstract

**Background:**

Concurrent chemoradiotherapy (CCRT) for abdominal cancer frequently induces muscle loss, weight loss, and malnutrition.

**Objective:**

This exploratory randomized phase II trial evaluated whether a multidisciplinary, mobile health (mHealth)–based multimodal rehabilitation program could preserve handgrip strength and muscle mass in patients with abdominal cancer undergoing CCRT.

**Methods:**

In this prospective, multicenter, randomized, open-label phase II trial (NCT05325554), 111 eligible patients with abdominal malignancies scheduled for CCRT were randomly assigned (1:1) to receive either multidisciplinary mHealth rehabilitation care (MRC; n=57) or standard care (SC; n=54). The MRC program was delivered by a dedicated multidisciplinary team using the AiNST mHealth platform and wearable heart rate monitors. The primary end point was handgrip strength at the end of CCRT (analyzed with analysis of covariance adjusting for baseline). Secondary end points were exploratory and analyzed without multiplicity adjustment; sensitivity analysis using false discovery rate (FDR) correction was performed.

**Results:**

Between February 2022 and April 2023, 111 patients were enrolled. Adherence was high (n=93, 83.9% achieved exercise targets). After adjusting for baseline handgrip strength, the MRC group had significantly higher handgrip strength at the end of CCRT than the SC group (adjusted mean difference 4.87 kg, 95% CI 3.36‐6.38; *P*<.001). Exploratory analyses of secondary end points (without multiplicity adjustment) showed that the MRC group also had better preservation of body weight (*P*=.005), skeletal muscle mass (*P*<.001), serum albumin (*P*=.009), prealbumin (*P*=.02), and lower rates of hematological toxicity (*P*<.05), as well as improved psychological status (distress thermometer [DT] and Hospital Anxiety and Depression Scale [HADS]) and nutritional scores (Nutritional Risk Screening 2002 [NRS-2002] and Patient-Generated Subjective Global Assessment [PG-SGA]) at the end of CCRT (all *P*<.05). All nominally significant secondary end points remained significant after FDR correction (*q*<.05). These findings are preliminary and should be interpreted with caution due to the open-label design, population heterogeneity, and exploratory secondary analyses.

**Conclusions:**

In this exploratory phase II trial, a multidisciplinary, mHealth-based multimodal rehabilitation program was associated with better preservation of handgrip strength, muscle mass, and nutritional status, as well as lower rates of certain treatment toxicities, compared with SC. However, definitive conclusions are limited by the open-label design, heterogeneity of tumor types, and short follow-up. Larger, blinded phase III trials are needed to confirm these findings.

## Introduction

Concurrent chemoradiotherapy (CCRT) is a cornerstone treatment for locally advanced abdominal malignancies, including gastric, pancreatic, and colorectal cancers [1]. While CCRT improves local tumor control and survival, it frequently induces debilitating adverse effects, including weight loss, malnutrition, fatigue, anxiety, depression, and bone marrow suppression [2-4]. Among these, chemoradiotherapy-induced muscle loss and decline in handgrip strength are particularly concerning: sarcopenia and reduced muscle strength independently predict poor treatment tolerance, lower quality of life, and worse survival [5-7].

The period of CCRT offers a critical window for intervention. Cancer prehabilitation and rehabilitation have emerged as promising strategies to mitigate these adverse effects [8,9]. Studies suggest that multimodal rehabilitation—combining exercise, nutrition, and psychological support—may outperform single-modality approaches [10,11]. However, randomized controlled trials of such comprehensive programs in patients with abdominal cancer undergoing CCRT remain scarce [12].

Patients receiving CCRT face additional barriers: nutrition and psychological support are not systematically integrated into routine oncology care, and access to multidisciplinary expertise is limited [13]. These gaps highlight the need for a structured, scalable model that delivers coordinated supportive care across multiple centers.

Mobile health (mHealth) technologies offer a promising solution. By providing real-time monitoring, personalized feedback, and remote communication, mHealth platforms can improve adherence and support data-driven decisions [14-18]. Several studies have demonstrated the feasibility of mHealth-supported interventions in cancer populations, including smartphone-based nutritional support in pancreatic cancer [19] and mHealth coaching to prevent muscle loss during neoadjuvant chemoradiotherapy for esophageal cancer [20]. Nevertheless, high-quality randomized trials of mHealth-facilitated, multidisciplinary rehabilitation in this population are lacking.

Handgrip strength was chosen as the primary end point, given its well-documented prognostic value for treatment tolerance and overall survival in patients with cancer [21], its responsiveness to multimodal interventions [22], and its recognition as a core outcome measure in clinical guidelines for sarcopenia and cachexia [23]. Accordingly, we conducted this prospective, multicenter, exploratory phase II trial to evaluate the feasibility and potential efficacy of a multidisciplinary, mHealth-based multimodal rehabilitation program in preserving handgrip strength and muscle mass in patients with abdominal cancer undergoing CCRT.

## Methods

### Trial Design and Participants

This prospective, investigator-initiated, multicenter, randomized, open-label phase II trial was conducted at 3 cancer centers in China: Zhongnan Hospital of Wuhan University, Renmin Hospital of Wuhan University, and Hubei Cancer Hospital. The study protocol was approved by the ethics committee of each participating hospital and registered with ClinicalTrials.gov (NCT05325554). Ethics approval was obtained in February 2022, and the first patient was enrolled in late February 2022. The registration record was released on March 8, 2022, after administrative verification by the registry. The study protocol was finalized and approved before any patient was enrolled, and no modifications were made to the registry entry after its release. The trial followed the CONSORT (Consolidated Standards of Reporting Trials) reporting guideline. Written informed consent was obtained from all patients.

The inclusion criteria were aged ≥18 years, a histologically confirmed abdominal malignant tumor scheduled for adjuvant or neoadjuvant CCRT, no distant metastasis, an Eastern Cooperative Oncology Group performance status of 0 to 1, adequate physical capacity for body composition analysis and 6-minute walk test, the ability to operate a smartphone, and adequate organ function.

The exclusion criteria were clinically significant cardiovascular disease; uncontrolled systemic disease (eg, poorly controlled diabetes); inability to swallow, chronic diarrhea, or intestinal obstruction; and neurological or psychiatric abnormalities affecting cognitive function.

The multimodal rehabilitation intervention started immediately after baseline assessment (within 3 days before CCRT initiation) and continued throughout the CCRT period (5‐6 weeks), with follow-up assessments at 4 weeks after CCRT.

### Sample Size Calculation

Sample size was based on the primary end point of handgrip strength at the end of CCRT. Using pilot data and published literature, we assumed a mean handgrip strength of approximately 25 (SD 6) kg in the control group. A between-group difference of 4 kg was considered clinically meaningful. With a 2-sided significance level of α=.05, 80% power, and a 15% dropout rate, we required 31 patients per group. To allow for subgroup analyses and ensure robustness, we planned to enroll 50 patients per group (total 100 patients). Due to a faster-than-anticipated recruitment rate and to further strengthen the power for subgroup analyses, enrollment was extended to 111 patients. Actual enrollment (111 patients) exceeded the target, providing >90% power for the observed effect size.

### Cancer Treatment

All patients received standard CCRT for 5 to 6 weeks. The synchronous chemotherapy regimen comprised 2 cycles of fluorouracil monotherapy or oxaliplatin combined with fluorouracil-based drugs. Radiotherapy was delivered using intensity-modulated radiotherapy with 6-MV X-rays, total dose 45 to 50.4 Gy in 25 to 28 fractions.

### Randomization and Masking

Randomization used a computer-generated sequence with a permuted block design (block size of 4 and concealed allocation) stratified by study center and primary tumor site. Outcome assessors for handgrip strength, body composition, and laboratory tests were blinded to group allocation. Due to the nature of the behavioral intervention, patients and treating clinicians could not be blinded (open label).

### Interventions

#### Standard Care Group

Patients received routine oncology care, including general dietary advice and symptom management, without structured rehabilitation interventions.

#### Multidisciplinary mHealth Rehabilitation Group

Patients received standard care (SC) plus a multidisciplinary, mHealth-based multimodal rehabilitation program.

### Multidisciplinary Team and Collaborative Workflow

The multidisciplinary team (MDT) consisted of radiation and medical oncologists (treatment decisions and safety monitoring), rehabilitation physicians (exercise prescription and progression), clinical nutritionists (individualized energy and protein targets and oral nutritional supplements), psychologists (stress management and coping skills), and trained research nurses (daily monitoring, data collection, patient communication, and early symptom detection).

The team met weekly to review each patient’s adherence, adverse events, and assessment results. Decisions on exercise adjustments, nutritional support intensification, or psychological referrals were made collaboratively and documented in the electronic case report form. This structured workflow ensured consistent, protocol-driven, personalized care.

### mHealth Platform and Wearable Device

The mHealth app AiNST (KanCare Nutrition) delivered the intervention. The app included exercise management, nutritional guidance, and health insights, with separate interfaces for patients and clinicians. Patients in the intervention group also wore a chest-strap heart rate monitor (XOSS) during exercise sessions, which provided real-time heart rate data synchronized with the app. The therapist dashboard allowed remote monitoring of adherence and intensity.

### Physical Intervention

All exercise sessions were supervised at hospital sports venues. Personalized exercise plans followed American College of Sports Medicine guidelines: three 50-minute aerobic sessions per week (5-min warm-up, 25-min moderate continuous exercise at 50%‐70% maximum heart rate, and 10-min cool-down) plus 20 minutes of resistance training targeting major muscle groups. The wearable device provided real-time heart rate feedback to guide exercise intensity.

### Nutritional Intervention

Patients received individualized nutritional counseling to achieve daily targets of 30 to 35 kcal/kg and 1.5 to 2.0 g protein/kg. Oral nutritional supplements (KanCare Nutrition) and whey protein were provided as needed. Daily monitoring via the app ensured goal achievement.

### Psychological Intervention

Psychological assessments used the distress thermometer (DT) and Hospital Anxiety and Depression Scale (HADS). Personalized counseling, relaxation techniques (breathing and meditation), and music therapy were provided via the app and the WeChat (Tencent) platform. Patients with severe distress were referred to mental health clinics.

### End Points and Assessment Schedule

The primary end point was handgrip strength (kg) at the end of CCRT, measured at baseline and at CCRT completion.

The secondary end points were body weight (kg), measured at baseline, weekly (twice per week), at CCRT completion, and at the 4-week follow-up; skeletal muscle mass (kg), assessed by bioelectrical impedance analysis at the same time points; laboratory parameters (hemoglobin, serum albumin, prealbumin, and absolute lymphocyte count), measured at baseline, weekly, at CCRT completion, and at the 4-week follow-up; nutritional status evaluated by Nutritional Risk Screening 2002 (NRS-2002) and Patient-Generated Subjective Global Assessment (PG-SGA) at baseline, weekly, at CCRT completion, and at the 4-week follow-up; psychological status assessed by DT and HADS at the same intervals; physical mobility, measured by the 6-minute walk test at baseline, at CCRT completion, and at the 4-week follow-up; adverse events, graded by the Common Terminology Criteria for Adverse Events version 5.0 and monitored continuously; and radiotherapy completion rate, progression-free survival, and overall survival.

### Systematic Tools for Multicenter Implementation

To maintain consistency across the 3 centers, we created a standardized protocol. All sites used the same mHealth app with identical algorithms. Before the trial began, every MDT member received centralized training. Coordinators held weekly virtual meetings to review adherence, and a shared data capture system synchronized patient assessments, adverse events, and intervention logs. These tools were intended to facilitate quality control and reduce intercenter variability, suggesting potential scalability. Further research is needed to confirm their feasibility in real-world settings.

### Statistical Analysis

#### Primary Outcome

The primary end point was handgrip strength at the end of CCRT. To compare the 2 groups while adjusting for baseline values, an analysis of covariance (ANCOVA) was performed with treatment group as the fixed factor and baseline handgrip strength as a covariate. The assumptions of linearity, normality of residuals, and homogeneity of regression slopes were verified.

#### Secondary Outcomes

All secondary end points were prespecified as exploratory. For continuous variables, after normality testing (Shapiro-Wilk), 2-sample *t* tests or Mann-Whitney *U* tests were used. Categorical variables were compared using chi-square tests or Fisher exact tests. No adjustment for multiple comparisons was applied to the primary analysis of secondary end points; therefore, results are reported as nominal *P* values and should be interpreted as hypothesis-generating. As a sensitivity analysis, Benjamini-Hochberg false discovery rate (FDR) correction was applied to all secondary end points. The software used was SPSS (version 25.0; IBM Corp) and R (version 4.3.1; R Foundation for Statistical Computing; *p.adjust* function).

### Ethical Considerations

We obtained written informed consent from all participants. The study was approved by the Ethics Committee of Zhongnan Hospital of Wuhan University (approval 2022015) and the respective ethics committees of all participating centers.

## Results

### Patient Characteristics

**Figure 1.**
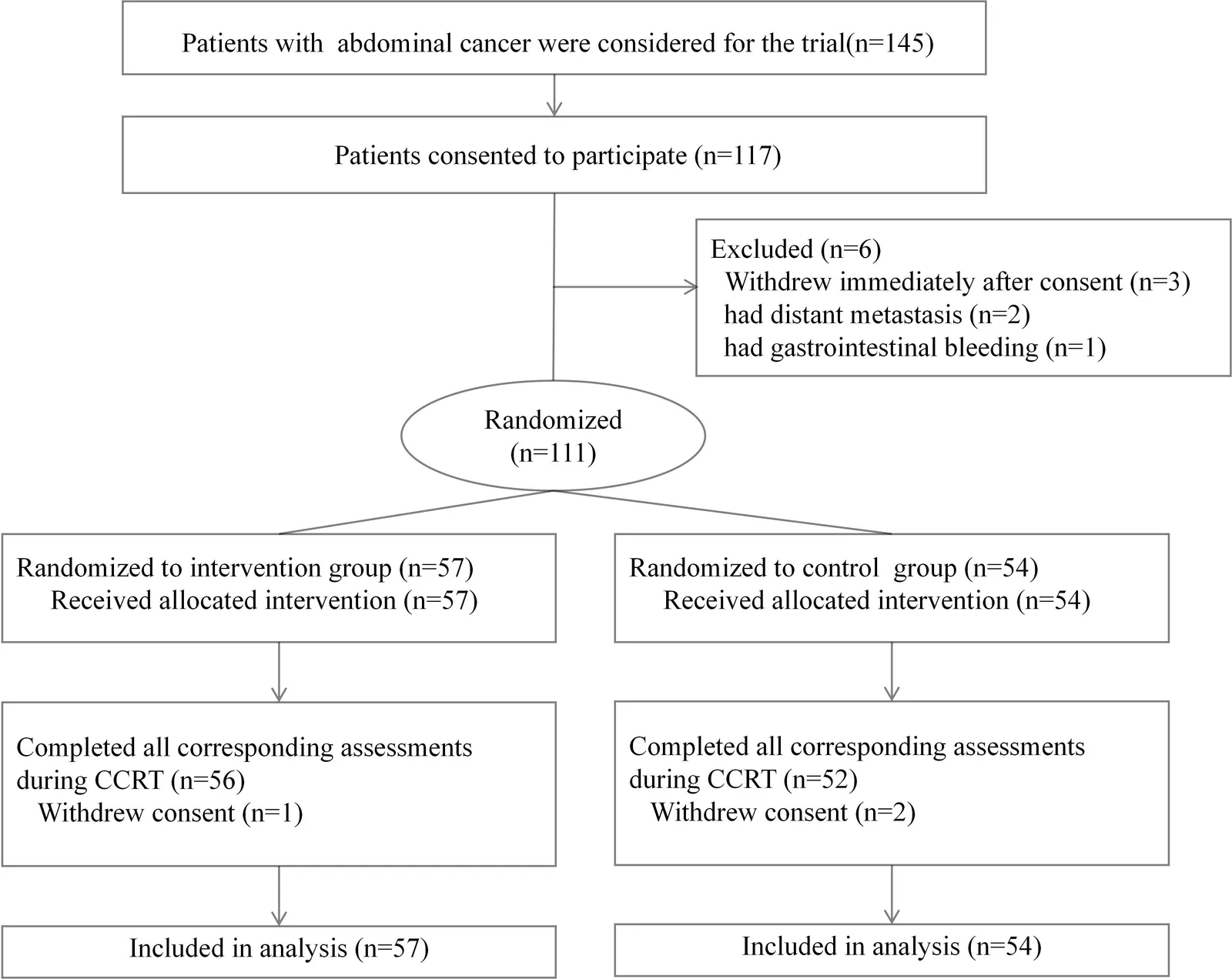
CONSORT (Consolidated Standards of Reporting Trials) flow diagram of patient enrollment, randomization, and follow-up. CCRT: concurrent chemoradiotherapy.

Between February 2022 and April 2023, we screened 188 patients, enrolled 111, and randomized them (multidisciplinary mHealth rehabilitation care [MRC]:n=57; SC: n=54 Figure 1). One MRC patient and 2 SC patients withdrew, leaving 108 for primary end point analysis. Baseline characteristics were well balanced between groups (Table 1). No statistically significant differences were observed.

**Table 1.** Baseline characteristics in patients^a^.

Characteristics	MRC^b^ (n=57)	SC^c^ (n=54)	*P* value
Age (y), mean (SD)	57.51 (11.09)	60.35 (7.45)	.12
Age (y), n (%)			
≥65	18 (31.6)	19 (35.2)	.74
<65	39 (68.4)	35 (64.8)	.74
Sex, n (%)			.14
Male	29 (50.9)	29 (53.7)	
Female	28 (49.1)	25 (46.3)	
Weight (kg), mean (SD)	60.26 (9.82)	62.60 (7.63)	.20
Hand grip strength (kg), mean (SD)	26.15 (7.54)	29.29 (7.97)	.06
Muscle mass (kg), mean (SD)	43.23 (8.68)	45.59 (6.60)	.24
BMI (kg/m^2^), mean (SD)	21.71 (2.29)	22.36 (2.52)	.16
PG-SGA^d^ score, mean (SD)	5.37 (3.13)	5.02 (2.95)	.57
NRS-2002^e^ score, mean (SD)	1.86 (1.12)	1.60 (0.99)	.24
6-min walk test, mean (SD)	479.36 (55.76)	467.89 (59.18)	.33
Distress thermometer, mean (SD)	2.58 (1.58)	2.55 (1.23)	.91
Hospital Anxiety and Depression Scale–Anxiety, mean (SD)	3.14 (1.57)	3.49 (1.42)	.3
Hospital Anxiety and Depression Scale–Depression, mean (SD)	3.20 (1.96)	3.50 (1.62)	.45
Hemoglobin (g/L), mean (SD)	118.51 (14.34)	124.00 (14.73)	.07
Albumin (g/L), mean (SD)	38.36 (3.95)	39.39 (3.52)	.18
Prealbumin (mg/L), mean (SD)	196.23 (56.99)	218.11 (45.05)	.06
Absolute lymphocyte count (10^9^/L), mean (SD)	1.32 (0.35)	1.48 (0.54)	.09
Elevated C-Reactive Protein, n (%)	2 (3.5)	1 (1.9)	>.99
Primary tumor site, n (%)			.98
Pelvic	40 (70.2)	38 (70.4)	
Upper Abdominal	17 (29.8)	16 (29.6)	
Purpose of radiotherapy			.70
Preoperative	11 (19.3)	12 (22.2)	
Postoperative	46 (80.7)	42 (77.8)	
Chemotherapy			.87
Fluorouracil monotherapy	35 (61.4)	34 (63)	
Fluorouracil plus oxaliplatin	22 (38.6)	20 (37)	
Radiation dose (Gy), mean (SD)	47.1 (2.9)	46.2 (2.5)	.10

a Percentages might not total 100% because of rounding. Categorical variables are presented as n (%), and continuous variables are presented as mean (SD). The Pearson chi-square test was used for categorical variables. The independent 2-sample *t* test was used for continuous variables.

b MRC: multidisciplinary mHealth rehabilitation care.

c SC: standard care.

d PG-SGA: Patient-Generated Subjective Global Assessment.

e NRS-2002: Nutritional Risk Screening 2002.

### Adherence to the Multidisciplinary mHealth Rehabilitation Program

In the MRC group (n=56), we planned a minimum of 15 exercise sessions. Patients attended on average 15.3 (SD 4.5) of 15 (102%) planned exercise sessions during treatment. Six patients completed more than 20 sessions, and 2 completed 25 sessions. The majority (n=47, 83.9%) achieved the target of 15 sessions, with very few (n=3, 5.4%) completing fewer than 5 sessions. No adverse effects related to the rehabilitation program were encountered.

### Primary Outcome

**Figure 2.**
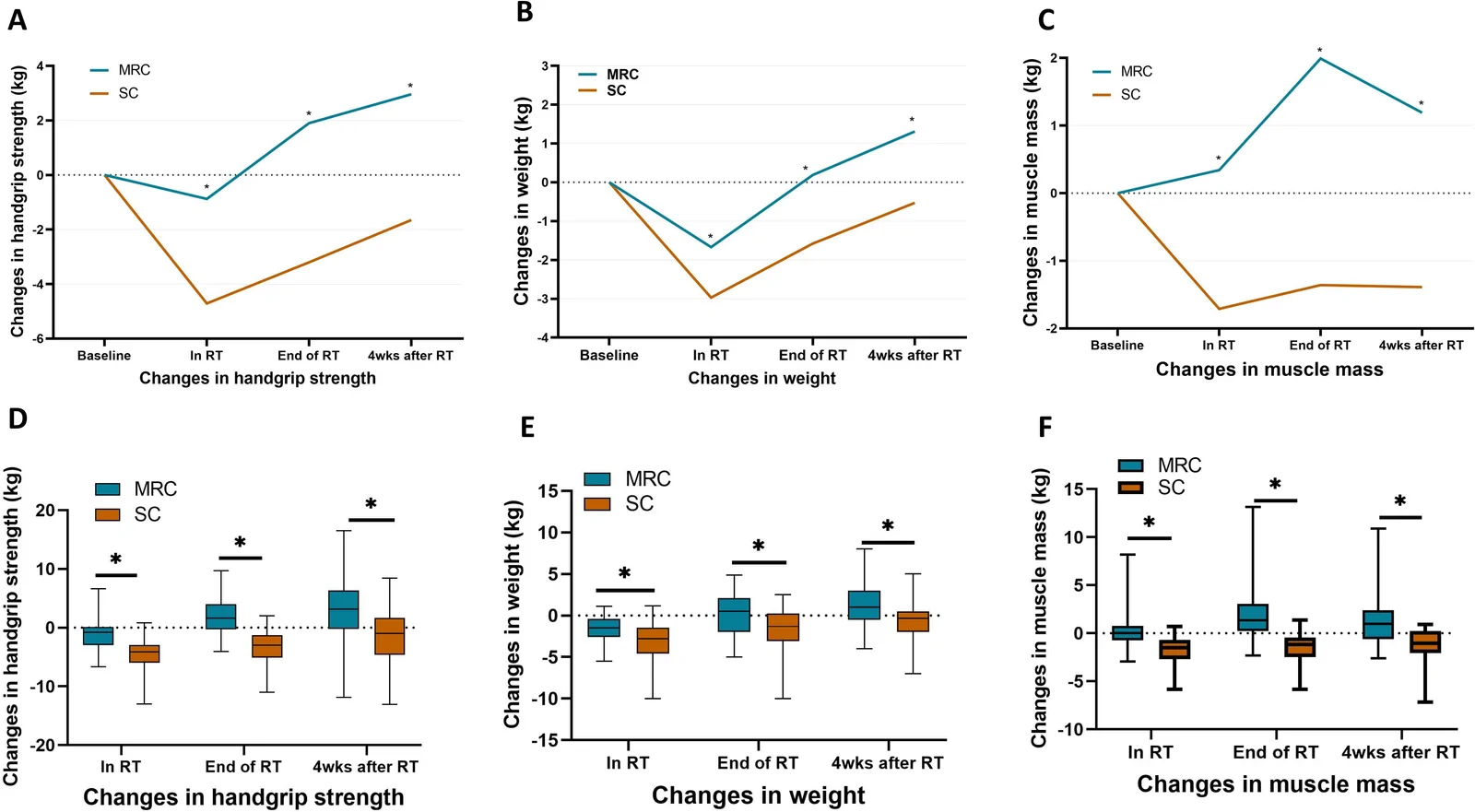
Changes in handgrip strength, body weight and muscle mass between multidisciplinary mHealth rehabilitation care (MRC) and standard care (SC) groups. Mean changes from baseline over time are shown in line graphs (A‑C): handgrip strength (A), body weight (B), muscle mass (C), assessed at baseline, during radiotherapy (In RT), end of radiotherapy (End of RT), and 4 weeks after radiotherapy (4wks after RT). Box‑and‑whisker plots (D‑F) show individual changes from baseline for handgrip strength (D), body weight (E), and muscle mass (F). *P<.05 for annotated time point.

The changes in handgrip strength from baseline are shown in Figure 2A and 2D. At the nadir during CCRT, the MRC group had a significantly smaller decline than the SC group (mean change –0.88, SD 3.09 kg vs –4.71, SD 2.83 kg, *P*<.001). At the end of CCRT, the MRC group showed a mean increase of +1.90 (SD 3.24) kg from baseline, while the SC group continued to decline (mean change –3.20, SD 3.19 kg, *P*<.001). At 4-week follow-up, the MRC group maintained a significantly higher handgrip strength (mean change +2.96, SD 5.96 kg vs –1.65, SD 5.09 kg, *P*<.001; Table S1 in Multimedia Appendix 1).

After adjusting for baseline handgrip strength, ANCOVA revealed a statistically significant difference between groups at the end of CCRT, favoring the MRC group. The adjusted mean difference was 4.87 kg (95% CI 3.36‐6.38; *P*<.001; partial η²=0.337). This result should be interpreted in the context of the open-label design and the exploratory nature of the trial.

### Secondary Outcomes

All secondary outcomes were analyzed without adjustment for multiple comparisons; therefore, the reported *P* values are nominal and the findings are considered exploratory. The following results should be interpreted as hypothesis-generating.

The MRC group experienced lower average body weight loss during CCRT compared with the SC group (mean 1.67, SD 1.51 vs mean 2.97, SD 2.54 kg; nominal *P*=.005; Table S1 in Multimedia Appendix 1), and by the end of CCRT, the MRC group had returned to baseline weight (mean weight change 0.19, SD 2.48 kg), whereas the SC group continued to lose weight (–1.58, SD 2.48 kg: nominal *P*=.002; Figure 2B and 2E). Fewer MRC patients lost >5% body weight (6/56, 10.7% vs 17/52, 32.7%; nominal *P*=.005). Muscle mass decline was also lower in the MRC group (mean change –0.8, SD 1.1 kg vs –2.1, SD 1.5 kg; nominal *P*<.001; Figure 2C and 2F). After FDR correction, all remained significant (*q*<.05).

**Figure 3.**
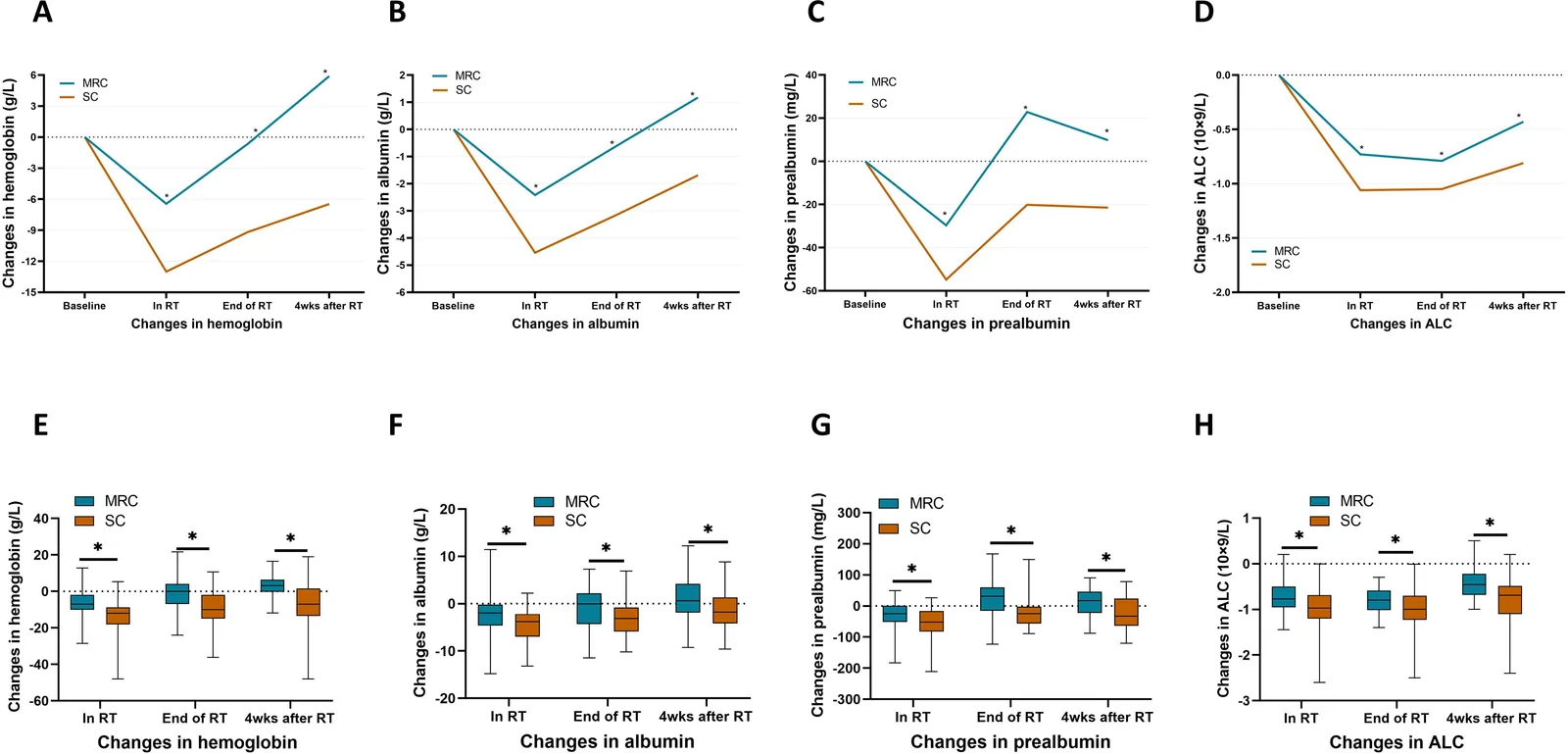
Changes in hemoglobin, serum albumin, prealbumin, and absolute lymphocyte count (ALC) between multidisciplinary mHealth rehabilitation care (MRC) and standard care (SC) groups. Mean changes from baseline over time are shown in line graphs (A‑D): hemoglobin (A), serum albumin (B), prealbumin (C), and absolute lymphocyte count (D), assessed at baseline, during radiotherapy (In RT), end of radiotherapy (End of RT), and 4 weeks after radiotherapy (4 wks after RT). Box‑and‑whisker plots (E‑H) show individual changes from baseline for hemoglobin (E), serum albumin (F), prealbumin (G), and ALC (H). *P<.05 for annotated time point.

The MRC group showed less decline in hemoglobin (mean 6.44, SD 7.62 vs mean 13.00, SD 9.21 g/L; nominal *P*<.001), serum albumin (mean 2.42, SD 4.32 vs mean 4.54, SD 3.26 g/L; nominal *P*=.009), and prealbumin (mean 29.67, SD 43.63 vs mean 54.83, SD 49.08 mg/L; nominal *P*=.02), and the proportion of patients with hemoglobin decrease >10% was higher in the SC group (19/52, 36.5% vs 6/56, 10.7%; nominal *P*=.001). Absolute lymphocyte count decline was milder in the MRC group at the 4-week follow-up (mean 0.43, SD 0.34 ×10⁹/L vs mean 0.81, SD 0.58 ×10⁹/L; nominal *P*=.001; Figure 3 and Table S2 in Multimedia Appendix 2); all remained significant after FDR correction.

**Figure 4.**
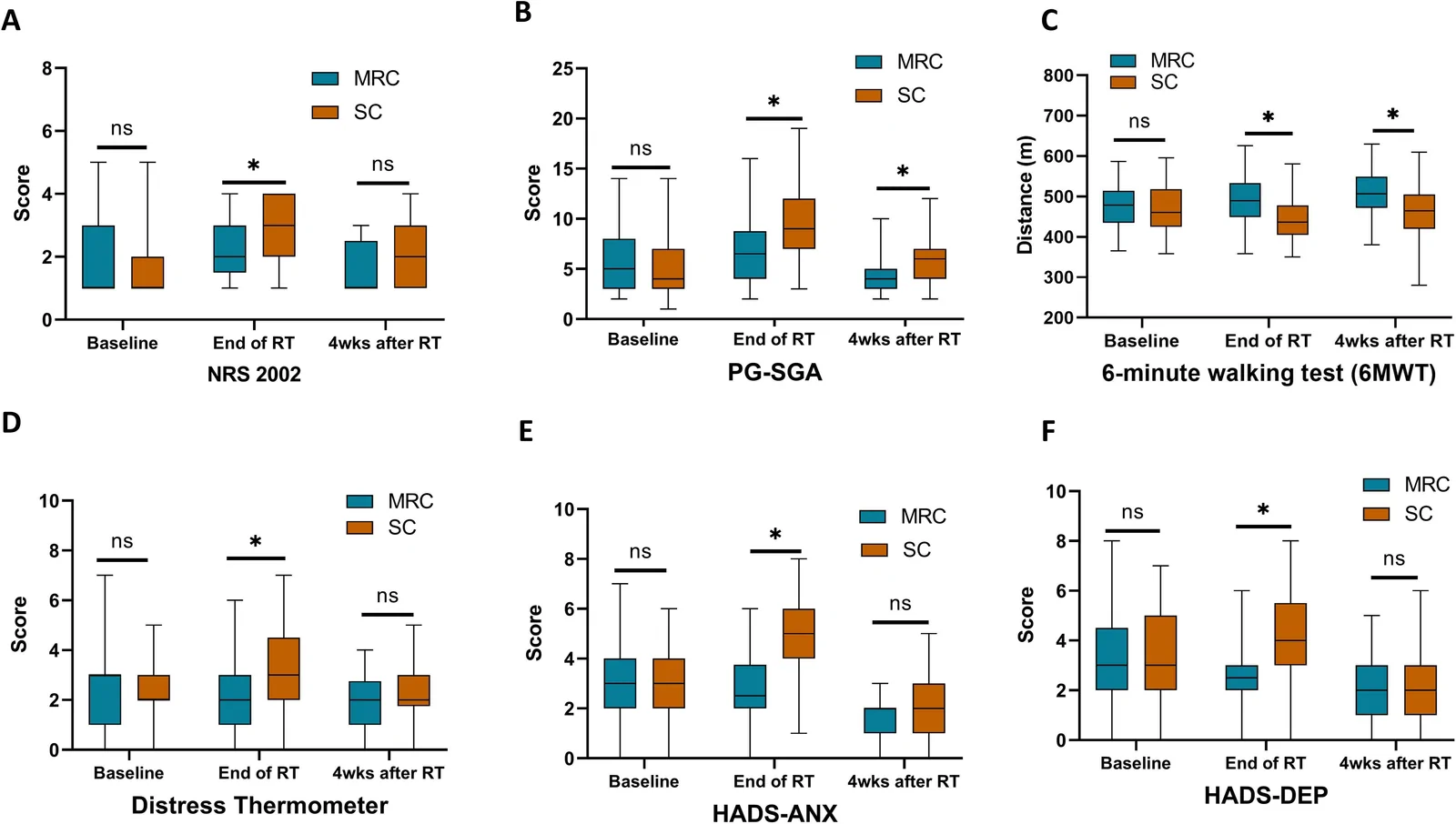
Secondary endpoints for the multidisciplinary mHealth rehabilitation care (MRC) and standard care (SC) groups assessed at baseline, end of radiotherapy (RT), and 4 weeks after RT. Box‑and‑whisker plots show individual distributions of (A) NRS 2002 nutritional risk screening score, (B) Patient‑Generated Subjective Global Assessment (PG‑SGA) score, (C) 6‑minute walking test (6MWT, distance in meters), (D) Distress Thermometer score, (E) Hospital Anxiety and Depression Scale‑Anxiety subscale (HADS‑ANX) score, and (F) Hospital Anxiety and Depression Scale‑Depression subscale (HADS‑DEP) score. *P<.05 for annotated time point.

At the end of CCRT, the MRC group showed significantly lower increases in average NRS-2002 and PG-SGA scores (both nominal *P*<.001; Figure 4A and 4B; Table S3 in Multimedia Appendix 3), maintained more stable 6-minute walk test performance (nominal *P*=.04; FDR-adjusted *q*=.046; Figure 4C; Table S3 in Multimedia Appendix 3), and had significantly lower DT, HADS-Anxiety, and HADS-Depression scores (all nominal *P*<.001; Figure 4D-4F; Table S3 in Multimedia Appendix 3), all of which remained significant after FDR correction.

The SC group experienced higher rates of grade 3 or higher leukopenia (10/52, 19.2% vs 3/56, 5.4%; nominal *P*=.04), grade 1 or higher thrombocytopenia (18/52, 34.6% vs 9/56, 16.1%; nominal *P*=.03), and elevated CRP (19/52, 36.5% vs 7/56, 12.5%; nominal *P*=.004); after FDR correction, all remained significant (*q*<.05). No significant differences were observed in radiotherapy interruption or completion rates (Table 2).

**Table 2.** Adverse events profile^a^.

Adverse event	MRC^b^ (n=56), n (%)	SC^c^ (n=52), n (%)	*P* value
Grade 3 or higher leukopenia	3 (5.4)	10 (19.2)	.04
Grade 1 or higher thrombocytopenia	9 (16.1)	18 (34.6)	.03
Elevated c-reactive protein	7 (12.5)	19 (36.5)	.004
Radiotherapy interruption rate	2 (3.6)	6 (11.5)	.15
Radiotherapy completion rate	56 (100)	50 (96.2)	.23
Weight loss ≥5%	6 (10.7)	17 (32.7)	.005
Hemoglobin decreased ≥10%	6 (10.7)	19 (36.5)	.001

a Percentages might not total 100% because of rounding. Categorical variables were presented by number (%). Pearson chi-square test for categorical data was used.

b MRC: multidisciplinary mHealth rehabilitation care.

c SC: standard care.

## Discussion

### Principal Findings

This multicenter randomized phase II trial suggests that a multidisciplinary, mHealth-based multimodal rehabilitation program can help preserve handgrip strength and muscle mass in patients with abdominal cancer undergoing CCRT. Compared with SC, the intervention significantly improved handgrip strength at the end of CCRT (primary end point). Exploratory analyses also indicated potential benefits for body weight, skeletal muscle mass, nutritional biomarkers, hematological toxicity, physical function, and psychological well-being. However, the open-label design, heterogeneity of the study population, lack of multiplicity adjustment for secondary outcomes (partly addressed by FDR sensitivity analysis), and short follow-up limit the strength of these conclusions.

Our MDT-based approach differed from traditional single-modality supportive care by simultaneously addressing physical, nutritional, and psychological declines. Weekly meetings, risk stratification, and shared decision-making helped ensure that no patient’s needs were overlooked. Multidisciplinary collaboration is increasingly recognized as a cornerstone of comprehensive cancer supportive care, especially during intensive treatments like CCRT. The high adherence rate (47/56, 83.9%) in our study further indicates that delivering such complex interventions with an organized MDT and digital tools is feasible.

The preservation of handgrip strength (5.10 kg difference; *P*<.001) and muscle mass (1.3 kg; *P*<.001) is clinically meaningful. Handgrip strength predicts treatment tolerance and survival in patients with cancer [5,21], and its decline during CCRT worsens outcomes. Our results show that a multimodal strategy combining exercise, nutrition, and psychological support—delivered via mHealth—can counteract the catabolic state induced by CCRT. Individual nutritional support [21,24] or exercise alone [25-27] has benefits, but combining multiple modalities likely produces a stronger anabolic effect. This aligns with previous reports on mHealth-based multimodal interventions in gastrointestinal cancers [19,20]. Nevertheless, due to the open-label design and the exploratory nature of the secondary analyses, these results require confirmation in future studies.

A key strength of our study is its multicenter design and the use of systematic tools to ensure consistent implementation across 3 centers. The unified mHealth platform, centralized training, and shared electronic data capture system were designed to facilitate quality control and reduce intercenter variability. This suggests potential scalability of the intervention, although the heterogeneous sample limits strong claims about generalizability.

Our results align with emerging evidence supporting mHealth and MDT interventions in cancer care. The SHINE-MDT trial demonstrated that multidisciplinary team support reduced radiotherapy interruptions and improved nutritional and psychological outcomes in patients with head and neck cancer [28]. Our study extends this concept to patients with abdominal cancer undergoing CCRT and adds mHealth-facilitated, real-time monitoring. The observed reduction in hematological toxicity—including lower rates of grade 3 or higher leukopenia and grade 1 or higher thrombocytopenia—represents a clinically meaningful finding, though direct comparisons are limited by differences in study design and populations.

At the end of CCRT, the intervention group showed significantly better psychological status than the control group. This finding suggests that the sense of empowerment from actively participating in one’s recovery, combined with supportive interactions with the MDT and direct benefits of exercise and relaxation techniques, may buffer the psychological burden of cancer treatment [29-31].

Several limitations should be acknowledged. First, the open-label design may have introduced performance bias, although outcome assessors for handgrip strength, body composition, and laboratory tests were blinded. Self-reported outcomes (psychological scores, PG-SGA, DT, and HADS) may be particularly susceptible to bias due to patient awareness of group assignment. Second, the study population was heterogeneous, including various abdominal cancer types, treatment intents, and chemotherapy regimens, which may limit generalizability. Third, the follow-up period was short (4 weeks after CCRT), preventing assessment of durability or survival effects. Fourth, the intervention was intensive and stopped after CCRT; the optimal duration remains unknown. Fifth, secondary end points were analyzed without multiplicity adjustment in the primary analysis; although FDR correction confirmed robustness, these results should be considered exploratory and hypothesis-generating. Sixth, the sample size was modest. Seventh, we did not use a risk-stratified intervention approach. Finally, cost-effectiveness was not assessed. Thus, these findings are preliminary and need confirmation in larger phase III trials with longer follow-up and prespecified multiplicity adjustments.

### Conclusions

This randomized phase II trial provides preliminary evidence that a multidisciplinary, mHealth-based multimodal rehabilitation program is feasible and may help preserve handgrip strength, muscle mass, and nutritional status, while also improving psychological well-being and reducing treatment toxicity in patients with abdominal cancer undergoing CCRT. However, due to the open-label design, population heterogeneity, and exploratory nature of the secondary analyses, these conclusions are limited and hypothesis-generating. Larger, well-powered phase III trials with blinded outcome assessment and longer follow-up are needed to confirm these findings before clinical implementation.

## Supplementary material

10.2196/98330 Multimedia Appendix 1 Mean changes from baseline to week 4 after concurrent chemoradiotherapy of the handgrip strength, body weight, and muscle mass.

10.2196/98330 Multimedia Appendix 2 Mean changes from baseline to week 4 after concurrent chemoradiotherapy for the multidisciplinary mHealth rehabilitation care and standard care group of the secondary end points (hemoglobin, serum albumin, prealbumin, and absolute lymphocyte count levels).

10.2196/98330 Multimedia Appendix 3 Mean changes from baseline to week 4 after concurrent chemoradiotherapy for the multidisciplinary mHealth rehabilitation care and standard care group of the secondary end points (psychological status, physical mobility, and psychological status).

10.2196/98330 Checklist 1 CONSORT checklist.

## Abbreviations

ANCOVA: analysis of covariance
CCRT: concurrent chemoradiotherapy
CONSORT: Consolidated Standards of Reporting Trials
DT: distress thermometer
FDR: false discovery rate
HADS: Hospital Anxiety and Depression Scale
MDT: multidisciplinary team
mHealth: mobile health
MRC: multidisciplinary mHealth rehabilitation care
NRS-2002: Nutritional Risk Screening 2002
PG-SGA: Patient-Generated Subjective Global Assessment
SC: standard care
