# Supplementary material for: Multidisciplinary mHealth Rehabilitation for Patients With Abdominal Cancer Who Are Receiving Chemoradiotherapy: Randomized Phase II Trial
Source: J Med Internet Res. 2026 Sep 8;28:e98330. doi: 10.2196/98330 (PMC13552644; doi:10.2196/98330)
Supplement: Supplementary file 2 [file jmir-v28-e98330-s001.docx]

**Supplementary Table 1.** Mean changes from baseline to week 4 after CCRT of the handgrip strength, body weight and muscle mass.

| **Characteristic** | | **MRC (n = 56)** | | **SC (n= 52)** | |  |
| --- | --- | --- | --- | --- | --- | --- |
|  |  | **Mean (±SD)** | **95%CI** | **Mean (±SD)** | **95%CI** | **p-value** |
| Change of hand grip strength, Kg | In RT | -0.88±3.09 | -1.78 to 0.08 | -4.71±2.83 | -5.61 to -3.84 | 0.000 |
|  | End of RT | 1.90±3.24 | 0.96 to 2.90 | -3.20±3.19 | -4.20 to -2.22 | 0.000 |
|  | 4wks after RT | 2.96±5.96 | 1.19 to 4.78 | -1.65±5.09 | -3.21 to -0.17 | 0.000 |
| Change of hand grip strength, Kg | In RT | -0.88±3.09 | -1.78 to 0.08 | -4.71±2.83 | -5.61 to -3.84 | 0.000 |
|  | End of RT | 1.90±3.24 | 0.96 to 2.90 | -3.20±3.19 | -4.20 to -2.22 | 0.000 |
|  | 4wks after RT | 2.96±5.96 | 1.19 to 4.78 | -1.65±5.09 | -3.21 to -0.17 | 0.000 |
| Change of muscle mass, Kg | In RT | 0.34±2.01 | -0.25 to 1.01 | -1.71±1.47 | -2.33 to -1.15 | 0.003 |
|  | End of RT | 1.99±2.83 | 1.11 to 2.96 | -1.36±1.50 | -1.98 to -0.74 | 0.000 |
|  | 4wks after RT | 1.19±2.88 | 0.18 to 2.33 | -1.39±1.90 | -2.33 to -0.68 | 0.001 |
