# Supplementary material for: Multidisciplinary mHealth Rehabilitation for Patients With Abdominal Cancer Who Are Receiving Chemoradiotherapy: Randomized Phase II Trial
Source: J Med Internet Res. 2026 Sep 8;28:e98330. doi: 10.2196/98330 (PMC13552644; doi:10.2196/98330)
Supplement: Supplementary file 3 [file jmir-v28-e98330-s002.docx]

**Supplementary Table 2.** Mean changes from baseline to week 4 after CCRT for the MRC and SC group of the secondary endpoints (hemoglobin, serum albumin, prealbumin, and absolute lymphocyte count levels).

| **Characteristic** | | **MRC (n = 57)** | | **SC (n= 54)** | |  |
| --- | --- | --- | --- | --- | --- | --- |
|  |  | **Mean (±SD)** | **95%CI** | **Mean±SD** | **95%CI** | **p-value** |
| Changes of hemoglobin, g/L | In RT | -6.44±7.62 | -8.62 to -4.05 | -13.00±9.21 | -15.68 to -10.57 | 0.000 |
|  | End of RT | -0.65±10.16 | -3.58 to 2.38 | -9.18±9.87 | -11.94 to -6.48 | 0.000 |
|  | 4wks after RT | 5.89±9.57 | 3.03 to 8.92 | -6.47±12.73 | -10.23 to -2.75 | 0.000 |
| Changes of albumin,g/L | In RT | -2.42±4.32 | -3.75 to -1.13 | -4.54±3.26 | -5.52 to -3.67 | 0.009 |
|  | End of RT | -0.61±4.33 | -1.97 to 0.72 | -3.15±3.75 | -4.16 to -2.05 | 0.003 |
|  | 4wks after RT | 1.17±4.54 | -0.17 to 2.52 | -1.69±4.24 | -2.86 to -0.60 | 0.002 |
| Changes of prealbumin,mg/L | In RT | -29.67±43.63 | -43.71 to -17.04 | -54.83±49.08 | -71.07 to -39.83 | 0.019 |
|  | End of RT | 22.83±59.06 | 4.22 to 40.65 | -20.15±47.91 | -34.27 to -5.17 | 0.001 |
|  | 4wks after RT | 9.74±45.68 | -3.53 to 24.73 | -21.49±50.11 | -36.16 to -8.17 | 0.004 |
| Changes of ALC, 10^9^/L | In RT | -0.73±0.33 | -0.82 to -0.63 | -1.06±0.54 | -1.21 to -0.91 | 0.001 |
|  | End of RT | -0.79±0.28 | -0.87 to -0.70 | -1.05±0.54 | -1.20 to -0.90 | 0.008 |
|  | 4wks after RT | -0.43±0.34 | -0.54 to -0.31 | -0.81±0.58 | -0.98 to -0.64 | 0.001 |
