# Supplementary material for: Multidisciplinary mHealth Rehabilitation for Patients With Abdominal Cancer Who Are Receiving Chemoradiotherapy: Randomized Phase II Trial
Source: J Med Internet Res. 2026 Sep 8;28:e98330. doi: 10.2196/98330 (PMC13552644; doi:10.2196/98330)
Supplement: Supplementary file 4 [file jmir-v28-e98330-s003.docx]

**Supplementary Table 3.** Mean changes from baseline to week 4 after CCRT for the MRC and SC group of the secondary endpoints (psychological status, physical mobility and psychological status).

| **Characteristic** | | **MRC (n = 57)** | | **SC (n= 54)** | |  |
| --- | --- | --- | --- | --- | --- | --- |
|  |  | **Mean (±SD)** | **95%CI** | **Mean (±SD)** | **95%CI** | **p-value** |
| NRS2002 | Baseline | 1.86±1.12 | 1.54 to 2.21 | 1.60±0.99 | 1.35 to 1.87 | 0.237 |
|  | End of RT | 2.31±1.04 | 2.02 to 2.59 | 2.83±1.07 | 2.56 to 3.13 | 0.014 |
|  | 4wks after RT | 1.71±0.84 | 1.51 to 1.95 | 1.85±0.91 | 1.61 to 2.12 | 0.440 |
| PG-SGA | Baseline | 5.37±3.13 | 4.47 to 6.20 | 5.02±2.95 | 4.16 to 5.91 | 0.574 |
|  | End of RT | 6.71±2.88 | 5.89 to 7.55 | 9.25±3.67 | 8.26 to 10.18 | 0.000 |
|  | 4wks after RT | 4.55±2.14 | 3.93 to 5.18 | 5.66±2.39 | 5.10 to 6.31 | 0.016 |
| DT | Baseline | 2.58±1.58 | 2.10 to 3.09 | 2.55±1.23 | 2.18 to 2.89 | 0.913 |
|  | End of RT | 2.45±1.49 | 2.00 to 2.91 | 3.39±1.77 | 2.83 to 3.94 | 0.010 |
|  | 4wks after RT | 1.80±0.93 | 1.50 to 2.08 | 2.05±1.08 | 1.74 to 2.38 | 0.249 |
| HADS-ANX | Baseline | 3.14±1.57 | 2.68 to 3.66 | 3.49±1.42 | 3.05 to 3.92 | 0.297 |
|  | End of RT | 2.72±1.43 | 2.30 to 3.19 | 4.63±1.70 | 4.11 to 5.20 | 0.000 |
|  | 4wks after RT | 1.70±0.90 | 1.43 to 1.95 | 2.12±1.38 | 1.70 to 2.56 | 0.101 |
| HADS-DEP | Baseline | 3.20±1.96 | 2.59 to 3.78 | 3.50±1.62 | 3.04 to 4.00 | 0.449 |
|  | End of RT | 2.59±1.37 | 2.20 to 3.00 | 4.15±1.80 | 3.61 to 4.68 | 0.000 |
|  | 4wks after RT | 1.84±1.29 | 1.47 to 2.24 | 2.37±1.58 | 1.89 to 2.87 | 0.096 |
| 6MWT, m | Baseline | 479.36±55.76 | 475.11 to 504.13 | 467.89±59.18 | 462.09 to 495.99 | 0.329 |
|  | End of RT | 498.33±64.34 | 479.40 to 519.14 | 446.04±59.25 | 430.40 to 463.32 | 0.000 |
|  | 4wks after RT | 510.84±55.75 | 493.72 to 527.21 | 461.44±61.24 | 444.41 to 477.50 | 0.000 |

Nominal p‑values are shown. After Benjamini‑Hochberg false discovery rate (FDR) correction, all originally significant secondary endpoints remained significant (q<0.05).
